# Improving Outcomes in Pancreatic Adenocarcinoma: A Systematic Review of Immunotherapy in Multimodal Treatment

**DOI:** 10.3390/medicina61061076

**Published:** 2025-06-11

**Authors:** Paul-Cristian Borz, Mihnea Bogdan Borz, Oliviu-Cristian Borz, Toader Zaharie, Claudia Hagiu, Lidia Munteanu, Simona Gurzu

**Affiliations:** 1 George Emil Palade University of Medicine, Pharmacy, Science and Technology of Targu-Mures, 540142 Targu Mures, Romania; dr.borzpaul@gmail.com (P.-C.B.); borz.m.bogdan@gmail.com (M.B.B.); drborzcristian@gmail.com (O.-C.B.); simona.gurzu@umfst.ro (S.G.); 2 Gastroenterology Department, Regional Institute of Gastroenterology and Hepatology Prof. Dr. Octavian Fodor, 400394 Cluj-Napoca, Romania; borz_paul96@yahoo.com (T.Z.); claudiahagiu@yahoo.com (C.H.); 3 Department of General Surgery, Targu Mures County Emergency Clinical Hospital, 540136 Targu Mures, Romania; 4 Department of Gastroenterology, Iuliu Hatieganu University of Medicine and Pharmacy, 400347 Cluj-Napoca, Romania; 5 Department of Pathology, George Emil Palade University of Medicine, Pharmacy, Science and Technology, 540012 Targu Mures, Romania

**Keywords:** immunotherapy, pancreatic adenocarcinoma, multimodal treatment

## Abstract

*Background*: Despite advances in chemotherapy and supportive care, pancreatic ductal adenocarcinoma (PDAC) continues to carry a dismal prognosis, with a five-year survival rate of approximately 13%. While immunotherapy has revolutionized treatment for several malignancies, its efficacy in PDAC remains limited. Recent research has shifted focus toward integrating immunotherapy with chemotherapy, radiation, and targeted therapies in an effort to overcome therapeutic resistance and improve outcomes. Ongoing clinical trials are actively investigating these multimodal strategies. *Materials and Methods*: A systematic search was conducted using PubMed and ScienceDirect to identify relevant studies published in the past six years. Search terms included “pancreatic adenocarcinoma immunotherapy,” “pancreatic cancer treatments,” and “combination treatments for pancreatic adenocarcinoma.” Only English-language articles were included. *Results*: A total of 126 articles were initially identified through the database search. After a full-text screening, 48 articles were deemed potentially relevant. Following a rigorous review, 11 studies met the inclusion criteria and were selected for analysis. These studies included randomized controlled trials, non-randomized controlled trials, and retrospective studies. Meta-analyses and case reports were excluded. Articles that failed to meet the inclusion criteria were excluded, primarily due to the absence of relevant data addressing the main objective of this review. *Conclusions*: Combination strategies with immunotherapy and chemotherapy offer modest survival gains in metastatic settings, yet efforts in resectable and borderline resectable disease have fallen short. These outcomes reflect the profound immunosuppressive forces of the PDAC microenvironment. A new era of treatment must move beyond broad immunotherapeutic applications toward a precision-driven model. Molecular markers, such as KRAS mutations and circulating tumor DNA (ctDNA) profiles, are beginning to illuminate paths for personalized therapy selection. Future progress will depend on biomarker-guided clinical trials, a deeper understanding of immune resistance mechanisms, and bold innovation at the intersection of immunology and tumor biology.

## 1. Introduction

Pancreatic ductal adenocarcinoma (PDAC), the most common form of pancreatic cancer, remains one of the most lethal malignancies worldwide. Despite advancements in chemotherapy and supportive care, the prognosis for PDAC remains extremely poor, with a five-year survival rate of approximately 13% [1,2]. Standard first-line chemotherapy regimens, such as FOLFIRINOX (Folinic acid, Irinotecan, Oxaliplatin, and 5-Fluorouracil) and Gemcitabine/Nab-Paclitaxel, provide only modest survival benefits. Unfortunately, resistance to cytotoxic chemotherapy severely limits long-term treatment success, and the underlying mechanisms remain largely unclear [3].

A major contributor to chemotherapy resistance in PDAC is the desmoplastic reaction, characterized by excessive collagen and extracellular matrix (ECM) deposition by pancreatic stellate cells. This results in a dense, fibrotic, and hypoxic tumor microenvironment that promotes tumor progression and metastasis while simultaneously impeding drug delivery. The desmoplastic stroma facilitates tumor resistance through pathways such as SPARC (Secreted Protein Acidic and Rich in Cysteine) and Hedgehog signaling, making it a histological hallmark of PDAC. Additionally, epithelial-to-mesenchymal transition (EMT) plays a critical role in chemoresistance by enabling tumor cells to acquire invasive and metastatic properties. EMT is associated with the downregulation of epithelial markers like E-cadherin and the upregulation of mesenchymal markers such as N-cadherin and Vimentin, and transcription factors including Snail, Slug, Twist, ZEB1, and ZEB2 [4,5]. Combination therapies aim to address these barriers by targeting both tumor cell plasticity and the tumor microenvironment. TGF-β inhibitors and MEK/PI3K pathway blockers help reverse EMT and enhance treatment sensitivity, while agents like PEGPH20, FAK inhibitors, and vitamin D analogs remodel the stroma to improve drug penetration. Dual-targeted strategies, such as CD40 agonists with chemotherapy or TGF-β blockades with immune checkpoint inhibitors, offer a promising approach to overcoming PDAC’s complex resistance mechanisms [6,7].

Immunotherapy has significantly transformed the treatment of several cancers, demonstrating remarkable success in advanced melanoma, non-small cell lung cancer, renal cell carcinoma, Hodgkin lymphoma, and several others. Checkpoint inhibitors targeting the programmed cell death protein 1 (PD-1) pathway have shown impressive results in these malignancies. However, their effectiveness in PDAC remains limited due to the highly immunosuppressive nature of the tumor microenvironment. This challenge highlights the necessity of developing multimodal therapeutic strategies to enhance treatment efficacy and improve patient outcomes [8].

PDAC is not solely driven by KRAS (Kirsten Rat Sarcom Viral Oncogene Homolog) mutations but also by alterations in three other key oncogenes—TP53, CDKN2A, and SMAD4—which collectively shape the tumor’s biology and contribute to its resistance to treatment. The RAS (Rat Sarcoma) protein functions as a central signaling hub, transmitting signals through a complex network of downstream effector pathways. It integrates inputs from various upstream sources, including receptor tyrosine kinases and other receptor complexes—such as those found on immune cells—all converging on RAS to promote cellular proliferation. The KRAS gene encodes a small GTPase that is frequently mutated in cancer, typically at one of three hotspot codons: 12 (most commonly), 13, or 61. These mutations result in constitutive activation of the KRAS protein, driving oncogenic signaling. The transforming potential of mutant KRAS is further amplified by co-occurring mutations, such as loss-of-function alterations in TP53, facilitating tumor growth and malignant progression [9].

Given the limited success of monotherapy in PDAC, recent research efforts have focused on combining immunotherapy with chemotherapy, radiation, and targeted therapies. Several clinical trials are currently investigating these combination strategies to overcome resistance and improve survival outcomes. A comprehensive systematic review is essential to assess the potential benefits of these emerging therapeutic approaches and to identify the most promising strategies for enhancing the treatment landscape for PDAC patients. This article explores recent advancements in combination therapy for PDAC and evaluates their clinical implications for improving patient survival.

## 2. Methods

### 2.1. Search Strategy

A systematic search of PubMed and ScienceDirect was performed to identify English-language studies published in the past six years. Keywords included “pancreatic adenocarcinoma immunotherapy,” “pancreatic cancer treatments,” and “combination treatments for pancreatic adenocarcinoma”. Only articles published in English were considered.

### 2.2. Eligibility Criteria

The included studies were prospective or retrospective clinical trials, either randomized or non-randomized, that evaluated immunotherapy combined with other treatments for pancreatic adenocarcinoma, with overall survival as a key outcome. Only studies published in English were considered. Case reports, case series, systematic reviews, and meta-analyses were excluded.

### 2.3. Selection and Data Collection Process

Two authors independently screened titles and abstracts for relevance. Full texts of eligible studies were reviewed, and key data were extracted for analysis.

Extracted data included the following: study title, first author, publication year, study design and phase, treatment type, number of participants, mean age, treatment arms, overall survival (OS), and progression-free survival (PFS).

## 3. Results

### Study Selection

**Figure 1:**
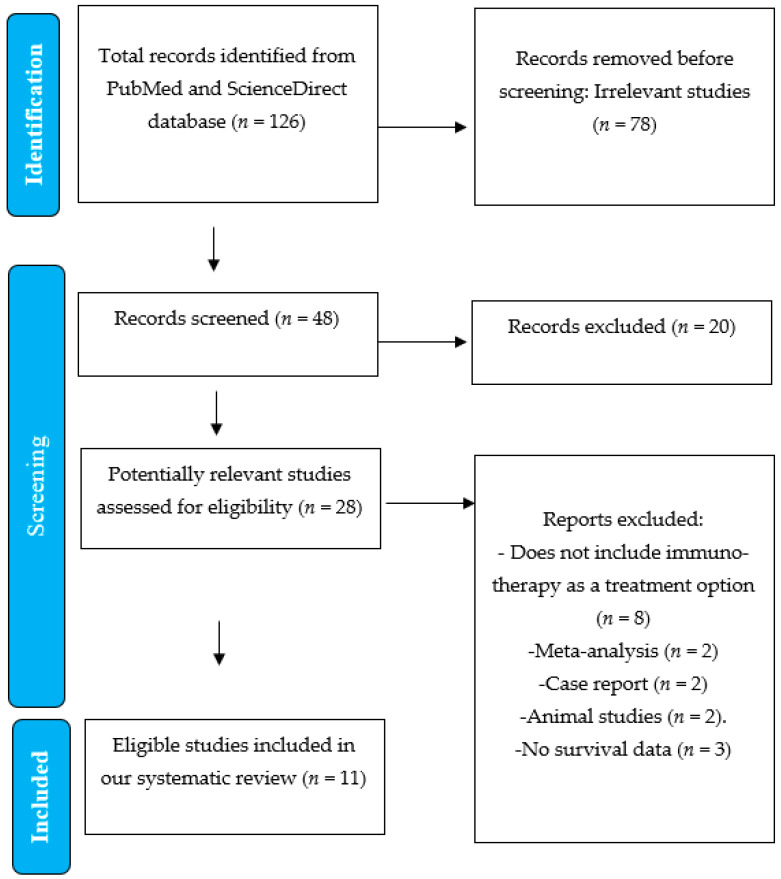
PRISMA flow chart of inclusion criteria.

A total of 126 articles were initially identified through the database search. After the full-text screening, 48 were considered potentially relevant. Following a detailed review, 11 studies met the inclusion criteria and were selected for analysis, as outlined in the PRISMA flow chart (Figure 1). These studies included randomized controlled trials, non-randomized controlled trials, and retrospective studies. Systematic reviews, meta-analyses, and case reports were excluded to avoid duplication of findings, as our objective was to independently synthesize and analyze primary data. Additionally, several clinical trials included in previous systematic reviews were also part of our selection, and their inclusion would have led to overlapping data and potential redundancy in the analysis. Articles were excluded primarily due to a lack of relevant data addressing the main objective of this review.

Articles were excluded if they lacked conclusive findings, demonstrated a non-adherence bias, or included patients with an Eastern Cooperative Oncology Group (ECOG) performance status greater than 3. This criterion was set to minimize confounding factors that could compromise the validity of survival rate analyses. Specifically, patients with an ECOG score above 3 were excluded due to their markedly poor prognosis, even with oncologic treatment. Additionally, the presence of significant comorbidities in this population increases the risk of non-cancer-related mortality, thereby introducing substantial bias in the assessment of treatment efficacy [10].

A firm exclusion criterion was the absence of immunotherapy as a treatment option for patients with PDAC. Additionally, studies that did not include data on overall survival were excluded.

The 11 selected studies consisted of the following:

- 1 retrospective study
- 6 randomized controlled trials
- 4 non-randomized controlled trials

These studies investigated the use of immunotherapy in combination with other treatment modalities (Table 1), including other types of immunotherapy, chemotherapy, CD40 inhibitors (Sotigalimab), pegylated recombinant human hyaluronidase (PEGPH20), Bruton’s tyrosine kinase inhibitors (Acalabrutinib), oncolytic viruses (*Pelareorep*), and high-affinity CXCR4 antagonists (Motixafortide/BL-8040). We did not include hazard ratios (HRs) and confidence intervals (CIs) in the main analysis because these data were inconsistently reported across studies. Some articles provided HRs and CIs, while others did not, and in certain cases, comparisons were made with historical controls rather than between randomized groups. Therefore, a meaningful statistical comparison across studies was not feasible. However, *p*-values were included in Table 1 when available.

In terms of study design, the trials were as follows:

- Two were Phase 1 trials, assessing safety and initial efficacy.
- Two were Phase 1/2 trials, evaluating safety, dosing, and preliminary therapeutic outcomes.
- Five were Phase 2 trials, focusing on treatment efficacy and adverse event profiles.
- One was a Phase 2/3 trial, designed to assess both efficacy and long-term clinical benefits in a larger patient cohort.
- One was a retrospective study, analyzing real-world treatment outcomes.

These selected studies provide a comprehensive overview of combination immunotherapy approaches and their potential clinical benefits.

## 4. Discussion

Despite advances in systemic therapy, outcomes for PDAC remain poor. Chemotherapy regimens like FOLFIRINOX and Gemcitabine/Nab-Paclitaxel provide only modest survival benefits. Although immunotherapy has transformed treatment in other cancers, its role in PDAC remains limited. This review examines recent clinical trial data on the efficacy of immunotherapy as part of multimodal treatment strategies for PDAC.

A single retrospective study included in this review compared immunotherapy plus chemotherapy versus chemotherapy alone in metastatic PDAC. Patients in the immunotherapy group received Nivolumab (*n* = 17), Pembrolizumab (*n* = 4), or Atezolizumab (*n* = 1), combined with various chemotherapy regimens. The chemotherapy-only group received Nab-Paclitaxel (*n* = 1), Nab-Paclitaxel plus Tegafur (*n* = 17), Gemcitabine (*n* = 2), Gemcitabine plus S-1 (*n* = 10), or Gemcitabine plus Cisplatin (*n* = 1).

The median overall survival (mOS) was significantly longer in the combination group (18.1 vs. 6.1 months; *p* = 0.021), and the median progression-free survival (PFS) was also improved (3.2 vs. 2.0 months). The safety profile was consistent with known immunotherapy toxicities, including hyponatremia (13.3%), thrombocytopenia (20%), neutropenia (46.7%), and elevated ALT (6.7%) [8,10,11].

We also reviewed a 2023 Phase 1b/2 RCT involving resectable or borderline resectable PDAC. Arm A received neoadjuvant Pembrolizumab plus chemoradiotherapy (CRT: Capecitabine and external-beam radiotherapy; *n* = 24), while Arm B received CRT alone (*n* = 13). Grade ≥ 3 adverse events occurred in 38% of Arm A and 31% of Arm B. Surgery was performed in 17 patients in Arm A and 7 in Arm B; the rest were ineligible due to progression or metastasis.

The mOS was 27.8 months in the combination group and 24.3 months in the CRT-only group; the median PFS was 18.2 vs. 14.1 months, respectively [12]. Despite slightly increased toxicity, the combination was well tolerated. However, no significant survival benefit or changes in CD8+ tumor-infiltrating lymphocytes were observed with Pembrolizumab.

Differences in outcomes likely reflect disease stage, with immunotherapy showing more benefit in metastatic PDAC. In contrast, CRT remains comparably effective and safer in resectable or borderline resectable cases.

Another therapeutic approach involves the combination of two immunotherapeutic agents with distinct mechanisms of action alongside chemotherapy. To evaluate this strategy, we included two studies in our review. The first study enrolled 180 patients with metastatic PDAC who were randomized into two arms. Arm A (*n* = 119) received chemotherapy (Gemcitabine and Nab-Paclitaxel) in combination with Durvalumab (a human IgG1 kappa monoclonal antibody targeting PD-L1) and Tremelimumab (an IgG2 monoclonal antibody targeting cytotoxic T-Lymphocyte-associated protein-3: CTLA-4). Arm B (*n* = 61) received chemotherapy alone. The mOS was 9.8 months in the combination arm and 8.8 months in the chemotherapy-alone arm. Median progression-free survival was 5.5 months and 5.4 months, respectively. These differences were not statistically significant (*p* = 0.72), indicating that the addition of dual checkpoint blockade did not confer a survival advantage in the unselected patient population [13].

Interestingly, a subgroup analysis revealed that patients harboring *KRAS* wildtype mutations experienced significantly prolonged survival in both treatment arms. In Arm A, patients with *KRAS* wildtype mutations had an mOS of 21.7 months compared to 8.8 months in patients with *KRAS* mutant tumors. Similarly, in Arm B, *KRAS*-mutant patients had an mOS of 14.9 months versus 7.8 months in those without the mutation. PFS was also significantly improved in *KRAS*-mutant patients, with *p* values of 0.007 in the combination arm and 0.02 in the chemotherapy arm [13,14]. Previous studies have shown that KRAS wild-type tumors, which comprise approximately 10–15% of PDAC, represent a distinct molecular entity compared to KRAS-mutant tumors. These tumors often harbor alternative oncogenic drivers, such as NRG1 gene fusions. However, research on the prognostic significance of KRAS mutation status remains limited, likely due to the relatively low prevalence of KRAS wild-type PDAC. Despite this, several studies involving tissue-based mutation profiling across both early-stage and metastatic cohorts have reported that patients with KRAS wild-type tumors tend to have better survival outcomes than those with KRAS mutations, suggesting a potential prognostic role for KRAS status.

These findings support KRAS mutation status as a potential biomarker for selecting patients who may benefit from immunotherapy, even in the absence of an overall benefit across the full cohort. Furthermore, the study highlighted the value of baseline circulating tumor DNA (ctDNA) sequencing to identify somatic mutations, supporting its potential utility in guiding personalized treatment strategies for metastatic PDAC in the future [15].

To further evaluate the potential benefit of adding chemotherapy to immunotherapy, we present a clinical trial that assessed the efficacy of combining two immune checkpoint inhibitors without chemotherapy in patients with metastatic PDAC. The study included two arms: Arm A consisted of 32 patients treated with a combination of Durvalumab (anti–PD-L1) and Tremelimumab (anti–CTLA-4), while Arm B included 32 patients who received Durvalumab monotherapy. Of the 64 patients enrolled, four discontinued treatment due to adverse events, including fatigue, diarrhea, pruritus, and hypothyroidism. Progression-free survival was 1.5 months in both arms. The mOS was 3.1 months in the combination group and 3.6 months in the monotherapy group, with no statistically significant difference in efficacy observed between the two treatment arms [16].

This underscores the limited benefit of PD-L1/CTLA-4 blockades as a monotherapy in this context. Future strategies to enhance checkpoint blockade efficacy may require additional immunomodulatory approaches, such as targeting antigen-presenting cells or incorporating other novel agents to stimulate a more robust anti-tumor immune response in PDAC [17].

The most extensively studied immunotherapy regimen in this review was the combination of a CD40 agonist antibody with chemotherapy. A randomized trial evaluated three arms: Arm A (Nivolumab + chemotherapy, *n* = 37), Arm B (Sotigalimab + chemotherapy, *n* = 31), and Arm C (Sotigalimab + Nivolumab + chemotherapy, *n* = 31). The mOS and PFS in Arm A were 16.7 and 6.4 months, respectively. Arm B showed an mOS of 11.4 months and a PFS of 7.3 months, while Arm C had an mOS of 10.1 months and a PFS of 6.7 months. Participants included patients with PDAC from stages I–IV, predominantly at stage IV (*n* = 80). Twelve patients discontinued therapy due to adverse events, physician decision, or non-compliance. No significant survival differences were observed between groups, and the addition of Sotigalimab did not improve outcomes [18].

Notably, in the Nivolumab–chemotherapy group, gene expression analysis revealed 15 signatures linked to survival. High expression of oxidative phosphorylation, fatty acid metabolism, xenobiotic metabolism, and bile acid metabolism genes correlated with better outcomes. Conversely, elevated TGF-β, TNF-α/NF-κB, and IL-6/JAK/STAT3 signaling was associated with poorer survival [18,19]. In the triplet arm, a lower frequency of activated CD38^+^ non-naïve CD8^+^ T cells was linked to prolonged survival, suggesting a role for immune profiling in guiding therapy.

To further evaluate Sotigalimab in metastatic PDAC, a separate randomized study assessed four arms: B1 (gemcitabine + nab-paclitaxel + Sotigalimab 0.1 mg/kg), B2 (same chemotherapy + Sotigalimab 0.3 mg/kg), A1 (chemotherapy + Nivolumab + Sotigalimab 0.1 mg/kg), and A2 (chemotherapy + Nivolumab + Sotigalimab 0.3 mg/kg). The mOS was 12.7 and 20.1 months in B1 and B2, respectively; the PFS was 12.5 and 10.4 months. In A1, the mOS was 15.9 months; the PFS in A1 and A2 was 10.8 and 12.4 months, respectively. OS for A2 was not reported [18,20].

All regimens were well tolerated, with mainly mild adverse events. The longest survival occurred in Arm B2, suggesting that Sotigalimab 0.3 mg/kg with chemotherapy may provide clinical benefit. However, adding Nivolumab did not enhance survival, indicating limited synergy between checkpoint blockades and CD40 agonism. These findings support the rationale for combining CD40 agonists with chemotherapy to activate innate and adaptive immunity in PDAC, particularly in metastatic settings. In contrast, a previous trial in borderline resectable PDAC showed no survival benefit from adding Sotigalimab, underscoring the importance of disease stage and patient selection in immunotherapy efficacy [20,21].

Only one additional study, aside from those previously mentioned, met the eligibility criteria for inclusion and evaluated patients with borderline resectable PDAC. This study included two treatment arms: one group received Acalabrutinib monotherapy (*n* = 35), and the other group received Acalabrutinib in combination with Pembrolizumab (*n* = 38). Acalabrutinib is a Bruton tyrosine kinase (BTK) inhibitor, and preclinical studies have suggested that combining BTK inhibition with a PD-1 blockade may enhance anti-tumor activity in PDAC. Among the 73 patients enrolled, 13 had stage I–III disease at baseline, while 58 patients had stage IV disease; disease stage information was unavailable for two patients. The mOS was 3.6 months for the Acalabrutinib monotherapy group and 3.8 months for the combination group. The median PFS was 1.4 months in both groups [22].

Although preclinical models demonstrated promising synergistic effects between BTK inhibition and PD-1 blockade, the clinical results did not replicate these findings. Both overall survival and progression-free survival remained very short in this patient population, highlighting the limited efficacy of this combination in advanced PDAC [22,23].

Motixafortide is a synthetic peptide that binds with high affinity to CXCR4, a G protein–coupled receptor, inhibiting the CXCR4–CXCL12 axis and enhancing T-cell infiltration into the tumor microenvironment. This mechanism increases tumor sensitivity to anti-PD-1 therapy, as shown in preclinical and clinical studies. A randomized trial evaluated Motixafortide in 39 patients with stage IV PDAC who had progressed after gemcitabine-based therapy. The second-line regimen included irinotecan, fluorouracil, folinic acid, Pembrolizumab, and Motixafortide. The mOS was 6.6 months, and the mPFS was 3.8 months. The combination showed clinical activity and was well tolerated in this poor-prognosis population. Liver metastases were present in 74.4% of patients, and those without liver involvement experienced longer survival, suggesting a potential predictive factor for response [24,25].

Another randomized trial assessed CXCR4 inhibition with Pembrolizumab, with or without chemotherapy, in metastatic PDAC. Group A received Pembrolizumab plus Motixafortide alone; Group B received the same agents with chemotherapy (*n* = 22). Group A was stratified into A1 (second-line, *n* = 16) and A2 (third-line or beyond, *n* = 21). The mOS was 7.5 months in A1 and 3.3 months in A2. Chemotherapy did not significantly improve survival in the second-line setting, but the A1 subgroup outperformed the standard second-line NAPOLI-1 regimen (mOS 6.1 months). Both trials included only patients who had progressed after first-line therapy. Further randomized studies are needed to evaluate whether CXCR4 inhibition combined with immunotherapy offers a greater benefit when introduced earlier in the treatment course [26,27].

A small clinical study involving 11 patients evaluated the potential benefit of combining Pelareorep (an oncolytic reovirus, specifically an isolate of the reovirus type II Dearing strain) with Pembrolizumab and chemotherapy (Irinotecan/Gemcitabine) as a second-line therapy. The mOS and PFS were 3.1 months and 2 months, respectively. Notably, three patients demonstrated a long-term benefit, with one achieving a partial response lasting 17.4 months and two maintaining stable disease for 4 months. Overall, the combination therapy was well tolerated and showed prolonged efficacy in a subset of patients, consistent with findings from preclinical studies. However, the majority of patients experienced poor overall survival, highlighting the need for further trials to better identify which patients may benefit from combination therapy with Pelareorep [28].

PEGPH20, a recombinant human hyaluronidase, was evaluated in combination with immunotherapy in patients with metastatic pancreatic ductal adenocarcinoma as a second-line treatment in the MORPHEUS study. In this trial, patients were randomized into two arms: the first arm received a combination of Atezolizumab and PEGPH20 (*n* = 66), while the second arm (control group) received chemotherapy consisting of Paclitaxel and Ramucirumab, a vascular endothelial growth factor receptor 2 (VEGFR2) inhibitor (*n* = 42). The mOS was 7.1 months in the combination therapy arm and 6.8 months in the control arm. The PFS was 1.5 months and 2.3 months, respectively. No significant differences in survival outcomes were observed between the two groups [29].

Although direct KRAS inhibitors have shown significant success in other cancers, the specific KRAS mutation variants that are prevalent in PDAC have proven largely resistant to such targeted approaches. As a result, current strategies are expanding beyond direct inhibition of the RAS protein to include targeting upstream regulators, downstream effectors, and the broader tumor microenvironment—including immune components influenced by aberrant RAS signaling. Additionally, mathematical modeling of resistance mechanisms has facilitated the development of innovative combination therapies aimed at enhancing the durability and effectiveness of targeted treatments [30].

In terms of regulatory status, it is important to note that Sotigalimab and Pelareorep have not received full FDA approval but have been granted an orphan drug designation, reflecting their potential for treating rare conditions despite the limited clinical data. PEGPH20 remains an investigational agent and has only been evaluated in early-phase clinical trials, with no current FDA approval. Acalabrutinib and Motixafortide are FDA-approved; however, their indications are restricted to hematologic malignancies and do not currently extend to pancreatic adenocarcinoma. All immunotherapeutic and chemotherapeutic agents discussed in this review are FDA-approved for oncologic use, underscoring the relevance and clinical applicability of the treatment strategies evaluated [31].

Emerging strategies aimed at reprogramming the pancreatic tumor microenvironment hold promise for overcoming current immunotherapy limitations. Advances in stromal remodeling agents, tumor vaccines, adoptive T cell therapies, and nanoparticle-based drug delivery systems are rapidly progressing from preclinical models to early-phase trials. In parallel, integrating high-throughput genomic and transcriptomic profiling into clinical trial design may allow for real-time adaptation of treatment protocols based on individual tumor biology. As our molecular understanding deepens, personalized immuno-oncology strategies tailored to tumor subtype, immune phenotype, and mutational burden may pave the way for a new standard of care in PDAC. Multi-institutional collaboration and biomarker-driven clinical trials will be critical in translating these innovations into tangible survival benefits.

### Limitations

The limitations of this systematic review primarily stem from the small sample sizes of the included trials. Many studies involved a limited number of patients, which restricts the generalizability of the findings. Additionally, some articles did not provide information on progression-free survival rates, either because these outcomes were not assessed as primary or secondary endpoints, or because the available data were insufficient for meaningful analysis. The limited number of eligible trials further restricts the ability to draw broad, evidence-based conclusions.

Most of the studies included in this review are Phase I or II clinical trials, primarily focused on metastatic PDAC. These trials are largely non-randomized, often with small sample sizes and heterogeneous patient populations, which limit the strength and generalizability of their conclusions. While they provide important preliminary insights into the safety and potential efficacy of immunotherapeutic approaches, the lack of robust comparative data underscores the need for well-designed, randomized Phase III trials. Future studies should aim to validate these early findings in larger, more uniform cohorts and explore biomarkers that could guide patient selection for immunotherapy. Overall, further clinical trials investigating immunotherapy within multimodal treatment strategies for pancreatic ductal adenocarcinoma are necessary to draw more definitive conclusions and to develop new therapeutic approaches aimed at improving survival rates in these patients.

**Table 1:** The main characteristics and results of the studies included in our systematic review.

	Stage of Cancer	Reference	Status of Clinical Trial	Study Phase	Immunotherapy + Chemotherapy Group	Chemotherapy Group	Number of Patients	Mean Age of Patients	Median Overall Survival, Months (95% CI); *p*-Value	Median Progression-Free Survival (95% CI); *p*-Value
1.	Metastatic	Ma J. et al. (2020) [8]	Completed	Retrospective	A = Pembrolizumab + Chemotherapy (*n* = 4); Nivolumab + Chemotherapy (*n* = 17); Atezolizumab + Chemotherapy (*n* = 1)	B = Chemotherapy Group (*n* = 36)	58	A = 56 B = 54	A = 18.1 months B = 6.1 months *p* = 0.021	A = 3.24 months B = 2.14 months *p* = 0.041
2.	Borderline Resectable and Metastatic	Padron L. J. et al. (2022) [18]	Abandoned	2	A = Nivolumab + Chemotherapy (*n* = 37) C = Sotigalimab + Chemotherapy + Nivolumab (*n*= 31)	B = Sotigalimab + Chemotherapy (*n* = 31)	105 (Stage I-III: *n* = 25 Stage IV: *n* = 80)	N/A	A = 16.7 months B = 11.4 months C = 10.1 months *p* = N/A	A = 8.1 months B = 7.3 months C = 6.7 months *p* = N/A
3.	Borderline Resectable PDAC	Katz M. H. G. et al. (2023) [12]	Abandoned	1b/2	A = Pembrolizumab + Chemotherapy (*n* = 24)	B = Chemotherapy (*n* = 13)	37 (Stage IA: *n* = 4 Stage IB: *n* = 12 Stage IIA: *n* = 8 Stage IIB: *n* = 6 Stage III: *n* = 7)	A = 66 B = 64	A = 27.8 months B = 24.3 months *p* = N/A	A = 18.2 months B = 14.1 months *p* = N/A
4.	Metastatic	Ko A. H. et al. (2023) [29]	Abandoned	1b/2	A = Atezolizumab +PEGPH20 (*n* = 71)	B = Chemotherapy (*n* = 46)	117	A = 60 B = 62	A = 7.1 months B = 6.8 months *p* = N/A	A = 1.5 months B = 2.3 months *p* = N/A
5.	Metastatic	O’Hara M. H. et al. (2021) [20]	Moved onto next phase	1b	A1 = Chemotherapy + Sotigalimab 0.1 mg/kg + Nivolumab (*n* = 6) A2 = Chemotherapy + Sotigalimab 0.3 mg/kg + Nivolumab (*n* = 6)	B1 = Chemotherapy + Sotigalimab 0.1 mg/kg (*n* = 6) B2 = Chemotherapy +Sotigalimab 0.3 mg/kg (*n* = 6)	24	A1 = 68 A2 = 63 B1 = 68 B2 = 61	A1 = 12.7 months A2 = 20.1 months B1 = 15.6 months B2 = Not estimable	A1 = 12.5 months A2 = 10.4 months B1 = 10.8 months B2 = 12.4 months *p* = N/A
6.	Metastatic	Renouf D. J. et al. (2022) [13]	Abandoned	2	A = Chemotherapy + Durvalumab + Tremelimumab (*n* = 119)	B = Chemotherapy (*n* = 61)	180	N/A	A = 9.8 months B = 8.8 months *p* = 0.72	A = 5.5 months B = 5.4 months *p* = 0.91
7.	Borderline to Metastatic	Michael O. et al. (2020) [22]	Abandoned	2	A = Acalabrutinib + Pembrolizumab (*n* = 38)	B = Acalabrutinib (*n* = 35)	73 (Stage I-III: *n* = 13 Stage IV: *n* = 58 No data: *n* = 2)	64	A = 3.6 months B = 3.8 months *p* = N/A	A = 1.4 months B = 1.4 months *p* = N/A
8.	Metastatic	Devalingam M. et al. (2020) [28]	Moved onto next phase	1b	A = Chemotherapy + Pelareorep + Pembrolizumab (*n* = 11)		11	64	A = 3.1 months	A = 2 months
9.	Metastatic	Bruno B. et al. (2021) [24]	Moved onto next phase	2	A = Chemotherapy + Motixafortide + Pembrolizumab		39	N/A	A = 6.6 months	A = 3.8 months
10.	Metastatic	O’Reilly E. M. et al. (2019) [16]	Abandoned	2	A = Durvalumab + Tremelimumab (*n* = 32) B = Durvalumab (*n* = 32)		64	65	A = 3.1 months B = 3.6 months *p* = N/A	A = 1.5 months B = 1.5 months *p* = N/A
11.	Metastatic	Bruno B. et al. (2020) [26]	Abandoned	2a	A = Bl-8040 + Pembrolizumab (*n* = 37) B = BL-8040 + Pembrolizumab + Chemotherapy (*n* = 22)		59	68	A1 = 3.3 months (third line therapy or beyond) A2 = 7.5 months (second line therapy B = 7.8 months *p* = N/A	N/A

## 5. Conclusions

Pancreatic ductal adenocarcinoma remains largely refractory to the success of immunotherapy seen in other cancers. While combination regimens with chemotherapy yield limited survival benefits in metastatic disease, similar strategies in resectable and borderline resectable settings have been disappointing. These outcomes underscore the formidable immunosuppressive nature of the PDAC tumor microenvironment.

Progress in PDAC immunotherapy requires a shift toward precision-based strategies. Emerging molecular markers—such as KRAS mutation status and circulating tumor DNA—offer promising avenues for patient stratification. Concurrently, innovative therapies aimed at disrupting stromal barriers, enhancing antigen presentation, and modulating immune cell infiltration are redefining the treatment landscape.

Future progress will depend on biomarker-driven clinical trials, a deeper mechanistic understanding of immune resistance, and translational research bridging immunology and tumor biology. Immunotherapy has the potential to alter the trajectory of PDAC—but only through strategies that effectively penetrate its biological defenses.

## Data Availability

No new data were created or analyzed in this study. Data sharing is not applicable to this article.

## Abbreviations

The following abbreviations are used in this manuscript

PDAC	Pancreatic ductal adenocarcinoma
ctDNA	Circulating tumor deoxyribonucleic acid
SPARC	Secreted Protein Acidic and Rich in Cysteine
KRAS	Kirsten Rat Sarcoma Viral Oncogene Homolog
RAS	Rat sarcoma
RCTs	Randomized controlled trials
ECM	Extracellular matrix
EMT	Epithelial-to-mesenchymal transition
PD-1	Checkpoint inhibitors targeting the programmed cell death protein 1
OS	Overall survival
PFS	Progression-free survival
PD-L1	Monoclonal antibody that targets programmed cell death ligand 1
CRT	Chemoradiotherapy
mOS	Median overall survival
BTK	Bruton tyrosine kinase
CTLA-4	Monoclonal antibody targeting cytotoxic T-Lymphocyte-associated protein-3
CXCR4	Chemokine receptor type 4
